# Discrepancies between Parent and Teacher Reports of Motor Competence in 5–10-Year-Old Children with and without Suspected Developmental Coordination Disorder

**DOI:** 10.3390/children8111028

**Published:** 2021-11-09

**Authors:** Li Ke, Anna L. Barnett, Yun Wang, Wen Duan, Jing Hua, Wenchong Du

**Affiliations:** 1 State Key Laboratory of Cognitive Neuroscience and Learning, Beijing Normal University, Beijing 100875, China; ke@bnu.edu.cn (L.K.); wangyun@bnu.edu.cn (Y.W.); 2 Centre for Psychological Research, Oxford Brookes University, Oxford OX3 0BP, UK; 3 Collaborative Innovation Centre of Assessment toward Basic Education Quality, Beijing Normal University, Beijing 100875, China; duanwen@bnu.edu.cn; 4 Department of Maternity and Children’s Health Care, Shanghai First Maternity and Infant Hospital, Tongji University School of Medicine, Shanghai 200092, China; jinghua@tongji.edu.cn; 5 NTU Psychology, Nottingham Trent University, Nottingham NG1 4FQ, UK

**Keywords:** parent report, teacher report, respondent discrepancies, motor competence, Developmental Coordination Disorder

## Abstract

Parents and teachers have knowledge of children’s daily motor performance yet may make different judgments about the levels of competence observed at home and school. The current study aimed to examine the discrepancies between parent and teacher reports using the Movement ABC-2 Checklist and the Developmental Coordination Disorder Questionnaire (DCDQ) on children with and without suspected Developmental Coordination Disorder (DCD). The Movement ABC-2 Test was administered to 1276 children aged 5–10 years in China. The Movement ABC-2 Checklist and DCDQ were completed by both parents and teachers of all children. A total of 172 children achieving a score below the 15th percentile on the Movement ABC-2 Test were identified as children with suspected DCD. Both parents and teachers showed suitable agreement in judging children’s motor competence but low sensitivity in identifying children with DCD. Parent scores of children’s motor competence were more closely associated with test performance scores compared to teacher scores. Teachers tended to over-rate children’s motor competence. The motor difficulties identified by parents were associated with low Movement ABC-2 Test scores on Manual Dexterity and Balance components, while motor difficulties identified by teachers were associated with the Balance component only. The results demonstrated discrepancies between parent and teacher reports, suggesting the importance of using a range of measures to identify and describe motor difficulties in children.

## 1. Introduction

Developmental Coordination Disorder (DCD) is characterized by difficulties in motor performance in daily activities that are not consistent with the child’s age and intelligence and cannot be explained by a medical or neurological condition or by intellectual impairment [1]. Studies have shown that DCD occurs in 1.4–19% of school-aged children, depending on the selection criteria used [2]. Previous studies have emphasized the importance of early identification of children with DCD, which is critical to facilitate intervention and prevent the development of emotional and social consequences of the disorder [3,4,5,6].

However, given the heterogeneous nature of the disorder, the identification of DCD is often a long process, and some children with motor difficulties are never diagnosed [7]. According to the DSM-V [1], DCD should be diagnosed based on the following criteria: (i) acquisition and execution of coordinated motor skills are below the expected level for age, given the opportunity for skill learning; (ii) motor skill difficulties significantly interfere with activities of daily living and impact academic/school productivity, prevocational and vocational activities, leisure, and play; (iii) onset is in the early developmental period; and (iv) motor skill difficulties are not better explained by intellectual or visual impairment or other neurological conditions that affect movement. A clinical assessment of motor proficiency is time-consuming and expensive, and a more practical approach is usually to use questionnaires as a first step in the assessment process to provide information on movement competence in everyday life settings (criteria i).

Currently, both parent and teacher questionnaires exist for the identification of DCD. The Movement ABC-2 Checklist (henceforth referred to as “the MABC-2-C”) [8] and the Developmental Coordination Disorder Questionnaire (henceforth referred to as “the DCDQ”) [9] are the two most widely used scales designed to help identify children who have everyday motor and coordination difficulties [10]. Both questionnaires were designed for use with children older than 5 years old, and both were developed for use by parents, teachers, or other professionals who have had regular contact with the child. Both include questions focusing on the individual’s motor performance (e.g., self-care, ball skills, etc.) that are related to functional daily living skills, and the respondent is required to provide a score on the child’s competence based on their observation and knowledge of the child. These scores are then summed to provide a total score. Both the MABC-2-C and DCDQ have been shown to have suitable psychometric properties and a suitable discriminative validity by identifying children with motor difficulties [11,12,13], and both have been widely used to identify children with motor difficulties who might require an assessment to confirm a diagnosis of DCD.

Although both questionnaires allow both parents and teachers to be the respondents of the information, research in other domains indicates that discrepancies often exist between parents’ and teachers’ judgment about the levels of competence observed at home and school. For example, a meta-analysis reviewed 119 studies and found that different informants’ (parents and teachers) ratings of social, emotional, and behavior problems in children are discrepant [14], and the discrepancies of different respondents have been found with a wide range of assessment methods used to assess abnormal behaviors in children, and a diversified ethnic and cultural backgrounds [15].

Parents and teachers can make different reports on children’s motor capabilities because of different reasons: first, parents and teachers usually interact with children in different settings (i.e., family and school), and certain motor behaviors may only occur in a specific environment. Teachers may have limited opportunities to observe children’s self-care activities, or fine motor skills if the class size is large, while parents may be less aware of their children’s classroom behaviors. Second, there may be different expectations of children’s motor behaviors by parents and teachers. Compared to parents, a teacher may have a better understanding and more realistic sense of what is expected of a typical child in the school context, as they have experience of working with many children. Parents may exaggerate differences between their children if they have more than one child because they have no other children with whom to compare [16]. Third, teachers may also have higher expectations for behavioral conformity given the structured setting at school, while parents who provide their children with unstructured time to play freely, so the focus on required tasks is not necessary. The above three reasons may lead to a systematic bias of parent and teacher ratings of a child’s motor competence.

To our knowledge, few studies have examined the discrepancies between parent and teacher reports of children’s motor impairment. There is no single measure or “gold standard” assessment to make a formal diagnosis of DCD, and the diagnosis of DCD requires a professional to make a judgment based on information from multiple sources and informants. Therefore, respondent discrepancies may cause confusion and have a significant impact on the accuracy of the assessment, identification, and intervention of children with motor difficulties.

Therefore, the aims of the current study were: (i). to investigate the discrepancies between parent and teacher reports using the MABC-2-C and the DCDQ on children with and without motor difficulties; (ii). to examine the concordance of parent and teacher scores with results from the Movement ABC-2 Test; and (iii). to examine the associations between scores from parents and teachers with children’s test performance in different motor domains.

## 2. Materials and Methods

### 2.1. Participants

The present study was part of a large national retrospective cohort study in China to explore motor development in Chinese children. A total of 1295 children aged 5–10 years from 51 nurseries and schools in 16 cities in China were recruited for the study. Data were missing for 19 children, and their data were excluded from the final analysis. All 1276 children were recruited from mainstream nurseries and schools. Children with severe visual, hearing, and intellectual impairments or those with severe developmental disorders who were required to attend special education schools/nurseries according to the local regulations were excluded in the current study. The numbers of children in each age and sex group are shown in Table 1.

### 2.2. Materials

The *Movement ABC-2 Checklist* has two main sections related to everyday movement skills. Section A, titled “Movement in a Static and/or Predictable Environment” (15 items), assesses three kinds of skills: Self-Care Skills, Classroom Skills, and Physical Education/Recreational Skills. Section B, titled “Movement in a Dynamic and/or Unpredictable Environment” (15 items), assesses three kinds of skills: Self-Care Skills, Ball Skills, and Physical Education/Recreational Skills. For all skills in Section A and Section B, respondents first decide if the child can perform the skill or not. If the child can perform the skill, they rate whether they can do it very well (0), or just ok (1); if the child cannot perform the skill, the respondent rates whether they can almost (2) do it or are not close (3). Thus, ratings vary along a 4-point ordinal scale. A raw score is obtained by summing the scores from all items (maximum score = 90). A higher score reflects greater difficulty. In its original instructions, two cutoff scores are provided for different age groups to represent the poorest 5% and 15% of scores based on the original normative data from teachers in the United Kingdon. Permission was obtained from the test publisher to translate the MABC-2-C and the test instructions from English into Chinese; common use of terms (e.g., the name and description of tasks in Chinese translation) from earlier publications in Chinese was used in the translation. A back-translation from Chinese to English was undertaken by independent translators, and the original and back-translated versions were equivalent in meaning. An expert panel with experts in relevant fields was convened to review the Chinese translation to make sure that the translation was appropriate to the local culture.

The *DCDQ* is a 15-item parent questionnaire instructing the respondents to rate the child’s motor proficiency compared with their peers of the same age. Questionnaire items include everyday activities such as playing ball (throwing, catching, hitting) and writing (fast, legibly, with appropriate effort/tension). Individual item scores are summed to give a total score from 15 to 75 (higher scores indicate better motor coordination), which is then used to determine if the score suggests the risk or absence of DCD. The cutoff scores of different age groups were determined by comparing the scores between a typically developing group and a DCD group with children from both Canada and England, and a more detailed explanation of how the cutoff scores were generated can be found in Wilson et al.’s paper [9]. The *DCDQ-Chinese translation* was used in the current study [12].

*The Test component (Age Band 1, AB1 and Age Band 2, AB2) of the Movement Assessment Battery for Children—2nd Edition-Chinese* [8,17,18] was used as an objective measurement of children’s motor performance. There are three motor domains involving eight tasks: 3 tasks measuring Manual Dexterity (posting coins or placing pegs; threading beads or lace; drawing); 2 tasks measuring ball skills (throwing/aiming and catching); and 3 tasks measuring balance (one-leg floor/board balance; walking along a line; jumping or hopping) (see Table 2). For each domain, a standard score was obtained based on the Chinese norms. A total score on the Movement ABC-2 Test was also obtained for each participant. Higher scores indicate better motor performance. Children who obtained a total standard score on the Movement ABC-2 Test below the 15th percentile were considered to have significant movement difficulties, in line with DCD.

### 2.3. Procedure

The Movement ABC-2 Test was conducted individually on each child by a trained assessor. All assessors had proficient experience in conducting psychological assessments with children in a similar age range. In all cases, children were assessed individually in their own nurseries/schools. The testing duration for each child was 30–40 min questionnaires, including the Movement ABC-2 Checklist and the DCDQ, were sent to all parents and teachers to complete.

Consent forms and instructions for distributing these for whole classes of children were delivered to participating nurseries and schools. Consent was obtained from both nurseries/schools and parents. Ethical approval was obtained by the Institutional Review Board (IRB) of the National Key Laboratory of Cognitive Neuroscience and learning, Beijing Normal University.

### 2.4. Data Analysis

Statistical comparisons were performed using paired-sample t-tests. Pearson correlations were conducted to examine the strength of correlations between parents’ and teachers’ scores on both MABC-2-C and DCDQ and the agreement between their scores with the Movement ABC-2 Test score. At last, linear regressions were conducted to examine which motor components as measured by the Movement ABC-2 Test can best explain the MABC-2-C and the DCDQ scores of parents and teachers. Data analysis was performed using IBM SPSS Statistics 25.

## 3. Results

172 (13.5%) children achieving a score below the 15th percentile on the Movement ABC-2 Test were identified as children “with suspected DCD” because the other three criteria as described earlier were not examined on the participants; the other 1104 children were identified as being “without suspected DCD” in the analysis.

First, parents’ and teachers’ scores on both scales were compared to each other using a paired-sample t-test. The results showed that parents and teachers generally provided similar scores on the MABC-2-C. However, on the DCDQ, teachers generally provided a higher score, indicating better motor performance both in children with and without motor difficulties (Table 3 and Table 4).

Second, in order to determine the agreement of the motor performance using the MABC-2-C and DCDQ by the parents and teachers, we used the Movement ABC-2 Test score as the objective measurement of children’s motor competence. Firstly, data from all participants were included in the analysis, and Pearson correlation was used to assess the agreement between the Movement ABC-2 Test score and the scores from the MABC-2-C and DCDQ by both parents and teachers. As shown in Table 5, all four scores from the MABC-2-C and DCDQ conducted by parents and teachers were significantly correlated to each other. They were also significantly correlated with the Movement ABC-2 Test scores. It should be noted that higher scores on the Movement ABC-2 Test and the DCDQ indicate better performance, while higher scores on the MABC-2 Checklist indicate poorer performance. This explains why the correlations with the MABC-2 Checklist were all negative, and all others were positive. The highest correlation was for parents on their DCDQ and MABC-2 C scores (−0.61) and for teachers on their DCDQ and MABC-2-C scores (−0.45). Correlations between the parents and teachers within and across the questionnaires were all 20–23. Although statistically significant, correlations between the questionnaires and the test were all below 0.2.

The correlations were also analyzed separately for each group of children, those with and without suspected DCD. For children with suspected DCD, the parent scores on the MABC-2-C and the DCDQ were significantly correlated with the Movement ABC-2 Test score (−0.31 and 0.27, respectively), while for teachers, only scores on the DCDQ were significantly correlated with the Movement ABC-2 Test score (0.24). For children without suspected DCD, only parent scores on the DCDQ were correlated with the Movement ABC-2 Test score, but no other significant correlation was found between parents’ or teachers’ scores and the Movement ABC-2 Test score. However, parents’ and teachers’ scores on both the MABC-2-C and DCDQ were significantly correlated with each other in both groups of children (Table 6 and Table 7).

The sensitivity of both questionnaires by parents and teachers defined by the percentage of children with suspected DCD (15% cutoff point with Movement ABC-2 Test) was also identified by the MABC-2-C and DCDQ. Both questionnaires have their own scoring system to identify children with DCD if the score is lower than a particular figure for different age groups. In this study, the sensitivity of parent and teacher reports from MABC-2-C and DCDQ was shown in Table 8 with respect to results from the MABC-2 Test.

Lastly, linear regression was conducted to examine which motor components as measured by the Movement ABC-2 Test can best explain the MABC-2-C and the DCDQ scores by parents and teachers. The standard scores of the three domains assessed by the Movement ABC-2 Test components (Manual Dexterity, MD; Aiming and Catching, AC; and Balance, BAL; more explanation of the tasks can be found in Table 2) were entered in a linear regression model as independent variables, to predict the total scores of both parent and teachers on the MABC-2-C and the DCDQ. Table 9 revealed that in children with motor difficulties, motor difficulties identified by the parents with the MABC-2-C and the DCDQ were mainly explained by Manual Dexterity and Balance competence as assessed by the Movement ABC-2 Test. Motor difficulties identified by the teachers with DCDQ were mainly explained by Balance competence. For children without motor difficulties, parents’ judgment was generally predicted by Manual Dexterity (with both the MABC-2-C and the DCDQ), and Aiming and Catching (only with the DCDQ); while teachers’ judgment was only associated with Manual Dexterity (only with the MABC-2-C) (Table 10).

## 4. Discussion

This study aimed to evaluate the consistency between parent and teacher reports on children’s motor competence. To our knowledge, this is the first population-based study to examine the discrepancies between parent and teacher reports on children’s motor competence with a benchmark of objective measurements. We compared parent and teacher scores on 5–10-year-old children in China with and without suspected DCD, and our results suggested that although both parents and teachers had suitable sensitivity in judging children’s motor competence, parents’ scores of children’s motor competence were more closely associated with motor test performance scores as measured by the Movement ABC-2 Test compared to teachers. Teachers tended to over-rate children’s motor competence. The motor difficulties identified by parents in children with suspected DCD were associated with low test scores on Manual Dexterity and Balance as measured by Movement ABC-2 Test, while motor difficulties identified by teachers were associated with Balance difficulties only.

Taking children with and without suspected DCD together, both parents’ and teachers’ scores on the MABC-2-C and DCDQ showed a significant correlation with each other and with children’s motor performance as assessed by the Movement ABC-2 Test. However, in children without suspected DCD, only parents’ scores in the DCDQ were significantly correlated with the children’s Movement ABC-2 Test score. While in children with motor difficulties, both parents (in the MABC-2-C and the DCDQ) and teachers (in the DCDQ) were significantly correlated with the children’s Movement ABC-2 Test score. These results suggest that with typically developing children, parent report with the MABC-2-C or teacher report with either the MABC-2-C or DCDQ do not provide valid information to discriminate different levels of their motor competence; but parents and teachers can provide suitable ratings in judging motor competence of children with motor difficulties. Previous studies on typically developing children reported weak-moderate correlation [19], or no correlation [20] between parents’ and teachers’ judgments and children’s motor performance as measured by objective motor assessments [19,20] with various assessment instruments. Our results were not surprising because the MABC-2-C and DCDQ as used in the current study were both designed to identify motor difficulties and may not be suitable to provide precise information on the movement competence of typically developing children.

Although there was an association with the Movement ABC-2 Test, the parents and teachers only showed a moderate sensitivity in identifying children with motor difficulties. The results were consistent with earlier studies that also reported a moderate discriminant power in identifying DCD in older children with the MABC-C or DCDQ. For example, Junaid, Harris, Fulmer, and Carswell [21] reported that the MABC checklist completed by teachers showed very low sensitivity in identifying DCD (14.3%). Schoemaker, Smits-Engelsman, and Jongmans [22] reported higher rates of sensitivity (between 50% and 80% at the 15th percentile cutpoint across different age groups). Therefore, as being previously suggested, results from questionnaires should be interpreted with caution, and the information derived from them is not enough to rely on alone for identifying children with motor difficulties from the general population [10].

The focus of the current study was to examine the consistency between parents and teachers on their scores of motor performance. Our results showed that scores of the parent and teacher with both questionnaires were significantly correlated. However, compared to teachers, parents’ judgment of children’s motor competence is more closely associated with test performance scores as measured by the Movement ABC-2 Test compared to teacher scores. The comparison between parent and teacher scores also suggested that teachers tended to provide a higher score, indicating better motor performance than parents, especially with the DCDQ. One of the explanations may be that teachers were busy professionals and may be less inclined to provide accurate information compared to parents who only need to focus on their own children. Another possible explanation of the results is that, compared to the DCDQ, the MABC-2-C includes more questions asking about self-care skills. Teachers may not have the opportunity to observe self-care skills such as “maintains balance while standing to pull on articles of clothing”, as asked in the MABC-2-C. In the current study, the teachers who were asked to complete the questionnaires were all the children’s key/charged teachers in the nursery/school, who usually have the most regular contact with the child, and who were also usually not the Physical Education/Sports teachers leading the outdoor activities with the children. That may explain why teachers’ scores in the DCDQ but not MABC-2-C showed a correlation with the Movement ABC-2 Test score in children with suspected DCD. The MABC-2-C does ask about classroom skills as well; however, most items in this section can also easily be observed in a non-classroom setting (e.g., manipulate small objects; form letters using a pen; use scissors to cut paper; walks around avoiding objects and persons; and transport objects around the room without dropping them). Therefore, compared to teachers, parent scores in both scales showed better agreement to the Movement ABC-2 Test in children with motor difficulties. However, it should be noted that, for teachers, even when they had to provide information on students in a relatively large amount, they still showed a fairly suitable validation with their judgments.

Furthermore, the regression analysis suggested that the motor problems identified by parents and teachers reflected children’s difficulties in different motor domains. The motor problems identified in children with motor difficulties by parents were mainly explained by motor difficulties in Manual Dexterity and Balance, while motor problems identified by teachers were mainly explained by children’s motor difficulties in Balance only. The results suggested that the teachers might not have much experience in observing children’s Aiming and Catching skills, which usually happen outdoors. In children with suspected DCD, the parent scores did not reflect children’s Aiming and Catching difficulties either; however, interestingly, parent scores of children without suspected DCD did reflect children’s Aiming and Catching skills. These results suggest that for children with suspected DCD, they may tend not to participate in activities requiring Aiming and Catching skills (e.g., throw beanbag, catch balls, hit balls, etc. as asked in both MABC-2-C and DCDQ); therefore, their parents may be unlikely to observe the children’s performance in those activities and provide valid judgments.

In the current study, we should consider the possibility that other conditions such as undiagnosed attention problems or other undiagnosed psychological or neuropsychological impairments of the children may affect the children’s performance in both the Movement ABC-2 Test and their parents and teachers’ reports on their daily movement performance, and not all children with poor movement performance (<15% in Movement ABC-2 Test in the current study) would be clinically diagnosed as DCD, but were considered as “suspected DCD” in the current study. However, the focus of the current study was the consistency between parents’ and teachers’ reports of children’s motor competencies, which can be considered as the first step to identify children with motor difficulties and DCD, and only children who can be recognized with potential motor difficulties by either their parents or teachers could possibly get the chance to be referred to clinicians for further assessment and diagnosis. One limitation of the current study is the use of the United Kingdom and Canadian norms for both scales, as Chinese norms were not available on either MABC-2-C or DCDQ. This may affect the sensitivity analysis as different movement development profiles between Chinese and British children have previously been reported [17]. However, all other analyses only used the raw scores of both scales, and the results shall not be affected. Further work is needed to develop the Chinese norm of both MABC-2-C and DCDQ, and the potential cultural differences behind the discrepancies should also be further examined. More importantly, for scales to assess children’s movement competencies, careful instructions should be provided on who the suitable respondents (parents, teachers, or other caregivers) are for the questionnaire and what potential issues may arise if different respondents were used.

In conclusion, our results showed that both parents and teachers had a suitable agreement in judging children’s motor competence but low sensitivity in identifying children with motor difficulties. Parent reports of children’s motor competence were more closely associated with test performance scores as measured by the Movement ABC-2 Test compared to teacher reports, and teachers tended to over-rate children’s motor competence. The motor difficulties identified by parents were associated with low scores on Manual Dexterity and Balance, while motor difficulties identified by teachers were associated with Balance only. The results demonstrated a lack of consistency between teacher and parent reports using the Movement ABC-2 Checklist and DCDQ, suggesting the importance of using a range of measures to identify a motor difficulty in children aged 5 to 10 years. Furthermore, parent and teacher questionnaires provide a different perspective and should be best combined with objective test performance measures as part of a broader assessment.

## Figures and Tables

**Table 1:** The number of children recruited in each age and sex group.

Age group (Year)	Boy	Girl	Total
5	120	112	232
6	103	94	197
7	118	100	218
8	111	99	210
9	116	100	216
10	109	94	203
Total	677	599	1276

**Table 2:** Tasks and measurements in the Movement ABC-2 Test.

Movement ABC-2 Test Motor Domain	Task-AB1	Task-AB2	Accuracy	Time	Actual Raw Score
Manual Dexterity (MD)	MD1 posting coins	MD1 placing pegs	√	√	Completion time
	MD2 threading beads	MD2 threading lace	√	√	Completion time
	MD3 drawing trail	MD3 drawing trail	√		Number of errors made
Aiming and Catching (AC)	AC1 catching beanbag	AC1 catching with two hands	√		Number of catches out of 10
	AC2 throwing beanbag onto mat	AC2 throwing beanbag onto mat	√		Number of catches out of 10
Balance (BAL)	BAL1 one-leg balance	BAL1 one-board balance	√	√	Time to keep balance as required
	BAL2 walking heels raised	BAL2 walking heel-to-toe forwards	√		Number of steps made as required
	BAL3 jumping on mats	BAL3 hopping on mats	√		Number of jumps/hops made as required

**Table 3:** Paired samples *t*-test of the MABC-2-C and the DCDQ between parents and teachers of children with suspected DCD (*n* = 172).

Test	Parents Mean (Min-Max, SD)	Teachers Mean (Min-Max, SD)	T-Value	*p*
MABC-2-C	10.88 (0–49; 10.31)	9.38 (0–43; 9.38)	0.2	0.84
DCDQ	58.03 (33–75; 9.66)	61.48 (23–75; 12.16)	−3.40	<0.001 ***

*** *p* < 0.001.

**Table 4:** Paired samples *t*-test of the MABC-2-C and the DCDQ between parents and teachers of children without suspected DCD (*n* = 1104).

Test	Parents Mean (Min-Max, SD)	Teachers Mean (Min, Max, SD)	T-Value	*p*
MABC-2-C	6.83 (0–55; 8.16)	9.89 (0–46, 9.71)	0.1	0.92
DCDQ	61.84 (15–75; 9.46)	64.54 (18–75, 9.88)	−7.05	<0.001 ***

*** *p* < 0.001.

**Table 5:** Pearson Correlations for Movement ABC-2 Test, and Movement ABC-2 Checklist and DCDQ scores completed by parents and teachers on all children (*n* = 1276).

Test	MABC-2-C-Parents	DCDQ-Parents	MABC-2-Teachers	DCDQ-Teachers
MABC-2-C-parents				
DCDQ-parents	−0.61 **			
MABC-2-C-teachers	0.20 **	−0.20 **		
DCDQ-teachers	−0.23 **	0.23 **	−0.45 **	
Movement ABC-2 Test	−0.17 **	0.16 **	−0.10 **	0.10 **

** *p* < 0.01.

**Table 6:** Correlation of Movement ABC-2 Test and the MABC-2-C and DCDQ scores completed by parents and teachers of children with suspected DCD (*n* = 172).

Test	MABC-2-C-Parents	DCDQ-Parents	MABC-2-C-Teachers	DCDQ-Teachers
MABC-2-C-parents				
DCDQ-parents	−0.70 **			
MABC-2-C-teachers	0.26 *	−0.22 *		
DCDQ-teachers	−0.29 **	0.35 **	−0.44 **	
Movement ABC-2 Test	−0.31 **	0.27 **	−0.06	0.24 **

* *p* < 0.05; ** *p* < 0.01.

**Table 7:** Correlation of Movement ABC-2 Test and the MABC-2-C and DCDQ scores completed by parents and teachers of children without suspected DCD (*n* = 1104).

Test	MABC-2-C-Parents	DCDQ-Parents	MABC-2-C-Teachers	DCDQ-Teachers
MABC-2-C-parents				
DCDQ-parents	−0.57 **			
MABC-2-C-teachers	0.16 **	−0.19 **		
DCDQ-teachers	−0.18 **	0.19 **	−0.45 **	
Movement ABC-2 Test	−0.05	0.08 *	−0.06	0.01

* *p* < 0.05; ** *p* < 0.01.

**Table 8:** The accuracy rate of parent and teacher reports from MABC-2-C and DCDQ to identify children with suspected DCD (*n* = 172).

	Parents		Teachers	
	MABC-2-C	DCDQ	MABC-2-C	DCDQ
Accuracy rate	35%	27%	28%	14%

**Table 9:** Linear regression models of how the scores of parents and teachers’ MABC-2-C and DCDQ reflect three different motor domains as assessed by the Movement ABC-2 Test on children with suspected DCD (*n* = 172).

	Respondent	Domain	*B*	*SE B*	*β*	*r*	*p*
Parents	MABC-2-C	MD	−1.23	0.33	−0.33	−3.78	<0.001 ***
		AC	0.13	0.34	0.03	0.40	0.69
		BAL	−0.90	0.30	−0.26	−3.03	0.003 **
	DCDQ	MD	0.91	0.30	0.26	3.01	0.003 **
		AC	0.39	0.32	0.11	1.24	0.22
		BAL	0.57	0.27	0.18	2.08	0.04 *
Teachers	MABC-2-C	MD	0.27	0.46	0.06	0.58	0.57
		AC	0.67	0.46	0.14	1.47	0.14
		BAL	−0.61	0.38	−0.16	−1.60	0.11
	DCDQ	MD	0.66	0.36	0.15	1.80	0.07
		AC	−0.27	0.38	−0.06	−0.72	0.48
		BAL	1.16	0.33	0.28	3.49	0.001 **

* *p* < 0.05; ** *p* < 0.01; *** *p* < 0.001.

**Table 10:** The linear regression models of how the scores of parents and teachers’ MABC-2-C and DCDQ reflect three different motor domains as assessed by the Movement ABC-2 Test children without suspected DCD (*n* = 1104).

	Respondent	Domain	*B*	*SE B*	*β*	*r*	*p*
Parents	MABC-2-C	MD	−0.27	0.12	−0.08	−2.30	0.02 *
		AC	−0.03	0.10	−0.01	−0.30	0.77
		BAL	0.10	0.14	0.02	0.69	0.49
	DCDQ	MD	0.28	0.13	0.07	2.16	0.03 *
		AC	0.25	0.11	0.08	2.33	0.02 *
		BAL	−0.07	0.16	−0.02	−0.46	0.65
Teachers	MABC-2-C	MD	−0.30	0.14	−0.07	−2.09	0.04 *
		AC	−0.07	0.12	−0.02	−0.62	0.54
		BAL	−0.10	0.17	−0.02	−0.56	0.58
	DCDQ	MD	0.18	0.13	0.04	1.33	0.18
		AC	0.05	0.11	0.01	0.41	0.69
		BAL	−0.14	0.16	−0.03	−0.88	0.38

* *p* < 0.05.

## Data Availability

The data that support the findings of this study are available from the corresponding author Wenchong Du upon reasonable request.
